# Influencing factors of physical activity among young and middle-aged patients with coronary heart disease: a multicenter cross-sectional study

**DOI:** 10.3389/fpubh.2026.1773456

**Published:** 2026-03-02

**Authors:** Mengying Yang, Jian Lin, Yingying Zheng, Hui Zhang, Peipei Yu

**Affiliations:** 1 Department of Cardiovascular Medicine, The Central Hospital of Wuhan, Tongji Medical College, Huazhong University of Science and Technology, Wuhan, Hubei, China; 2 Key Laboratory for Molecular Diagnosis of Hubei Province, The Central Hospital of Wuhan, Tongji Medical College, Huazhong University of Science and Technology, Wuhan, Hubei, China

**Keywords:** adult, coronary disease, cross-sectional studies, exercise, middle-aged, risk factors

## Abstract

**Objective:**

To investigate the level of physical activity (PA) and influencing factors among young and middle-aged patients with coronary heart disease (CHD).

**Methods:**

A cross-sectional survey was conducted among 326 young and middle-aged patients with CHD in four tertiary hospitals in Hubei Province. This study adopted the general information questionnaire, International Physical Activity Questionnaire-Long (IPAQ-L), Fear of Activity in Patients with Coronary Artery Disease (Fact-CAD), Exercise Self-Efficacy Scale (ESES), Family Adaptation, Partnership, Growth, Affection, Resolve index (APGAR), The Piper Fatigue Scale-12 (PFS-12), and Patient Health Questionnaire-9 items (PHQ-9) as assessment tools. According to whether the level of PA met the recommended standard of the guideline, the participants were divided into the qualified group (*n* = 112) and the non-qualified group (*n* = 214). Univariate analysis and binary logistic regression analysis were performed using SPSS 25.0 to summarize the influencing factors of patients' PA.

**Results:**

The rate of PA compliance among young and middle-aged patients with CHD was 34.36%. Binary logistic regression analysis showed that hemoglobin, glycated hemoglobin, fear of movement, exercise self-efficacy, family care, and fatigue were the influencing factors of PA among young and middle-aged patients with CHD (*P* < 0.05).

**Conclusion:**

Young and middle-aged patients with CHD generally exhibit low levels of PA, a result of combined physiological, psychological, and social factors. Healthcare professionals should develop multi-dimensional intervention programmes to improve the level of patients' PA, thereby achieving cardiac rehabilitation.

## 1 Introduction

Cardiovascular diseases pose a severe global public health threat (1), and China faces an especially prominent burden in this context. According to domestic and international data, there are 330 million individuals living with cardiovascular diseases in China, including over 11.39 million cases of coronary heart disease (CHD) (2). A particularly alarming trend is the rapid rise among young and middle-aged patients with CHD, who account for 26.37% to 36.28% of all CHD cases in China, with an annual incidence growth rate of 6.37% to 20.47% (3). This rapid increase in the incidence rate and mortality rate (4), coupled with the long-term treatment and rehabilitation needs of these patients, imposes substantial medical, economic, and social burdens on Chinese families and society, making targeted prevention and rehabilitation interventions for this group increasingly urgent (5).

Physical activity (PA) is widely recognized as a core component of CHD management, with a well-documented dose-response relationship with cardiovascular mortality (6, 7). American Heart Association, European Society of Cardiology, and Chinese guidelines for secondary prevention of cardiovascular disease all emphasize the importance of PA for the rehabilitation of CHD patients (8–11). The European Society of Cardiology recommends that patients with CHD engage in at least 150–300 min of moderate-intensity PA or 75–150 min of high-intensity PA per week (12). A study in South Korea showed that moderate to high intensity PA reduced the risk of cardiovascular events in patients with CHD, making it an important strategy for improving their prognosis (7).

Notably, insufficient PA among patients with CHD is a consistent global challenge, and Chinese data further underscore the urgency of addressing this issue locally. A survey of 1,162 CHD patients in China showed that only 40% of CHD patients were able to maintain regular PA 12 months after discharge, with walking being their main PA. Thirty-seven percent of patients reported no intention to engage in regular PA (13). This situation aligns with international findings: South Korean follow-up research involving over 130,000 cardiovascular patients and Italian longitudinal studies have similarly confirmed that a large proportion of patients experience PA or fail to maintain PA (14, 15). This cross-country consistency highlights the universality of the PA inadequacy problem. Thus, it is necessary to investigate the factors influencing PA of these patients, in order to provide a basis for the subsequent formulation of targeted intervention strategies.

Although existing studies have explored the influencing factors of PA in CHD patients, there are research deficiencies in targeting young and middle-aged patients with CHD. First, existing studies tend to focus on older adults and general CHD populations, while ignoring the unique characteristics of young and middle-aged patients as a special group. Second, existing studies only focus on single-dimensional factors and lack systematic research that integrates physiological, psychological, and social factors to construct a comprehensive influencing factor model. Additionally, most of the existing studies are single-center studies with limited representativeness.

Notably, Compared with older adult patients, young and middle-aged patients with CHD have distinct characteristics: they are not only the main undertakers of family economy and important pillars of social productivity but also shoulder the multiple responsibilities of caring for children and supporting older adults (16, 17). This special social role makes young and middle-aged patients bear enormous psychological pressure and social role conflicts after illness, which poses higher challenges to their disease management and rehabilitation compliance.

In this study, the age range for young and middle-aged patients with CHD was defined as 18 to 59 years old. This definition was determined based on previous relevant research (18, 19). Therefore, this study aims to explore the influence of physiological factors, psychological factors and social factors on the PA levels of young and middle-aged patients with CHD through multicenter research. The results of this research will provide an important theoretical basis for clinical practice. By identifying the core influencing factors, healthcare professionals can systematically assess, thereby designing targeted PA promotion programs.

## 2 Materials and methods

### 2.1 Study design and participants

This study adopted a cross-sectional design and recruited young and middle-aged patients with CHD who were hospitalized in the department of cardiovascular medicine of four tertiary hospitals in Hubei Province from July 2025 to September 2025. The inclusion criteria for participation in this study were as follows: (1) individuals aged between 18 and 59 years; (2) individuals diagnosed with CHD confirmed by coronary angiography; (3) a minimum CHD duration of 3 months; (4) individuals who were conscious, free of communication barriers, and possessed adequate comprehension and expressive abilities; (5) individuals who voluntarily consented to participate in the study. Exclusion criteria were as follows: (1) individuals with severe heart failure, respiratory failure, malignant tumors, or other severe conditions; (2) individuals with other diseases that impair PA; (3) individuals who had received blood transfusion, intravenous iron therapy, or had adjusted their hypoglycemic drugs (including types and dosages) within the past 3 months. The sample size was 10 to 15 times that of the independent variables. A total of 22 independent variables were included in this study, so the required sample size was calculated to be at least 220.

### 2.2 Measures

#### 2.2.1 General information questionnaire

Compiled by researchers based on literature review, including gender, age, educational level, marital status, residence situation, monthly per capita household income, course of the disease, heart function classification, etc.

#### 2.2.2 International Physical Activity Questionnaire-Long (IPAQ-L)

This questionnaire was developed by International Consensus Group on Physical Activity Measurement to survey respondents‘ PA over the past week, including five dimensions: work, transportation, housework, leisure, and sedentary behavior, with a total of 27 items. The questionnaire classifies individuals' PA levels into low, medium, and high based on the duration of PA of varying intensity and weekly energy expenditure from PA. This scale has a test-retest reliability of 0.75 and good reliability and validity. It is currently used in 12 countries around the world to collect public exercise and health data (20, 21).

#### 2.2.3 Fear of Activity in Patients with Coronary Artery Disease (Fact-CAD)

Fact-CAD is a unidimensional scale developed by Ozyemisci-Taskiran et al. (22) based on literature reviews, qualitative interviews, and expert reviews, through the collection and analysis of quantitative and qualitative data. It consists of 21 items. Each item uses a 5-point Likert scale, with scores ranging from 0 to 4, corresponding to “never” to “always,” respectively. The total score ranges from 0 to 84 points, with some items scored in reverse. A higher total score indicates a higher level of exercise fear in patients. The Cronbach's α coefficient of the scale is 0.92, and the test-retest reliability is 0.89, making it a suitable tool for assessing the severity of exercise fear in patients with CHD. The time required to complete the scale is approximately 4–7 min.

#### 2.2.4 Exercise Self-Efficacy Scale (ESES)

This scale was developed by Bandura (23) and translated into Chinese by Tung et al. (24). It is used to assess individuals' judgments regarding their self-performance and ability to achieve success in challenging sports environments, serving as an effective measure of sports beliefs. The scale consists of 18 items, with each item scored on a scale of 0 to 100, where 0 indicates “very little confidence,” 50 indicates “moderate confidence,” and 100 indicates “very high confidence.” The total score is calculated by summing the scores of all items and dividing by the total number of items. A higher score indicates a higher level of exercise self-efficacy in patients. The Cronbach's α coefficient for this scale is 0.963.

#### 2.2.5 Family Adaptation, Partnership, Growth, Affection, Resolve index (APGAR)

This scale was developed by Smilkstein et al. (25) and consists of five aspects: family adaptability, partnership, growth, affection, and resolve. It uses the Likert three-level scoring method, with each aspect scored from 0 to 2 points, and the total score ranges from 0 to 10 points. The higher the score, the more satisfied the individual is with family functions. The Cronbach's α coefficient of this scale is 0.840 and the validity index is 0.800.

#### 2.2.6 The Piper Fatigue Scale-12 (PFS-12)

This scale was developed and revised by Piper et al. (26) and localized into Chinese by Qiao et al. (27). PFS-12 is a self-rating scale used to assess the current fatigue status of patients, covering four dimensions: behavior, emotion, sensation, and cognition, with a total of 12 items. A numerical scoring method ranging from 0 to 10 points is adopted, all of which are positive scores. The score is calculated as the sum of all item scores divided by the total number of items or the number of items within that dimension. The higher the score, the more severe the fatigue. The reliability of the total scale is 0.92.

#### 2.2.7 Patient Health Questionnaire-9 items (PHQ-9)

PHQ-9 was developed based on the nine diagnostic criteria for depressive disorder outlined in the Diagnostic and Statistical Manual of Mental Disorders, Fourth Edition (DSM-IV). Although it comprises only half the number of items found in many other depression measurement tools, it demonstrates good sensitivity and specificity. It can both assist clinical diagnosis and assess the severity of depressive symptoms (28). In 2009, Bian et al. (29) evaluated the questionnaire among 600 outpatients at a general hospital. Results indicated a Cronbach's alpha coefficient of 0.857, test-retest reliability of 0.947, sensitivity of 0.91, specificity of 0.97, and a Kappa value of 0.884, demonstrating sound reliability and validity (29).

### 2.3 Data collection

Researchers conducted on-site surveys to collect data. After obtaining approval from the relevant department of the hospital, they conducted a questionnaire survey among young and middle-aged patients with CHD in the cardiovascular medicine department using standardized instructions. They explained the purpose of the study, confidentiality principles, and completion methods, ensuring informed consent from patients before collecting data. If patients had low literacy levels and could not read the text, the researchers assisted them by asking questions and recording their answers to help complete the questionnaire. After data collection was completed, two graduate students entered the data independently and simultaneously.

### 2.4 Assessment of PA and quality control of data

All investigators received systematic training on the use of IPAQ-L, including the standard explanation of each activity item, the guidance methods for respondents to recall, and the identification of inconsistent answers. Investigators guided the respondents to recall their PA in the past 7 days with the help of daily activity scenarios (such as commuting, work, housework, sports) to reduce memory bias. After collecting the data, we screened the IPAQ-L scores. For extreme values, we rechecked with the corresponding respondents to confirm the accuracy of the data. After data collection was completed, two graduate students entered the data simultaneously. Following entry, the data were compared, and any discrepancies were resolved by rechecking the original questionnaire and re-entering the data to ensure accuracy and reliability.

### 2.5 Statistical methods

Data analysis was performed using SPSS 26.0 statistical software. For continuous variables that followed a normal distribution, data were expressed as (x ± s) and analyzed using *t*-tests; for those that did not follow a normal distribution, data were expressed as [M (P_25_, P_75_)] and analyzed using rank-sum tests. For categorical variables, data were expressed as frequency (percentage) [*n* (%)] and analyzed using chi-square tests. Univariate and binary logistic regression analyses were performed with PA attainment as the dependent variable. *P* < 0.05 was considered statistically significant. To assess whether the model exhibits multicollinearity, we calculated the variance inflation factor (VIF) and tolerance. VIF values exceeding 10 or tolerance values below 0.1 were taken as the criterion for identifying severe multicollinearity.

### 2.6 Ethical considerations

This study was approved by the ethics committee of the research institution, with the approval number: WHZXKYL2025-161. Before the formal questionnaire survey, investigators first assessed the patient's current physical condition (such as whether there is chest pain, shortness of breath, and other discomfort in the recent week) and obtained the consent of the attending physician to confirm that the patient is in a stable condition and suitable for completing the questionnaire. The total time required to complete all questionnaires was controlled within 30 min, and the survey was conducted in a quiet and comfortable environment (such as the ward or outpatient rest area) to avoid the patient's fatigue. Each survey site was equipped with a first-aid kit and a professional nurse on standby. If a patient experienced chest pain, dizziness, palpitations, or other health issues during the survey, the survey was immediately stopped, and the nurse gives first-aid treatment according to the situation, and contacts the attending physician for further processing. After the patient's condition stabilizes, the survey is decided whether to continue or terminate according to the doctor's advice. These measures ensure that the data collection process conforms to the principle of non-maleficence.

## 3 Results

This study aimed to investigate the level of PA and its influencing factors among young and middle-aged patients with CHD, and the following are the detailed results. A total of 340 questionnaires were distributed. After eliminating the invalid ones, 326 valid questionnaires were retrieved, with an effective recovery rate of 95.88%.

### 3.1 Characteristics of participants

The general information and disease-related details of 326 young and middle-aged patients with CHD are shown in Table 1. Among them, there were 247 males and 79 females. 93.25% of the patients were married. 32.82% of the patients have a junior high school education. 91.10% of the patients lived with their families. The average monthly income per household member is between 2,000 and 5,000 yuan, accounting for 43.56%. 62.58% of the patients had a disease course of 1 to 5 years. Most patients were classified as grade I in cardiac function (62.27%) (see Table 1).

**Table 1:** Characteristics of participants (*n* = 326).

**Variables**	**Number of cases (*n*)**	**Proportion (%)**
**Gender**		
Male	247	75.77%
Female	79	24.23%
**Religious belief**		
Yes	5	1.53%
No	321	98.47%
**Marital status**		
Unmarried	8	2.45%
Married	304	93.25%
Divorce	10	3.07%
Widowed	4	1.23%
**Educational level**		
Primary school and below	26	7.98%
Junior high school	107	32.82%
High school/Technical secondary school	98	30.06%
Junior college	49	15.03%
Bachelor's degree or above	46	14.11%
**Residence situation**		
Living alone	29	8.90%
Living with families	297	91.10%
**The location of the family**		
Rural area	21	6.44%
County town	24	7.36%
City	281	86.20%
**Monthly per capita household income (Yuan)**		
<2000	28	8.59%
2000–5000	142	43.56%
5001–10000	126	38.65%
>10000	30	9.20%
**Course of the disease**		
<1 year	99	30.37%
1–5 years	204	62.58%
>5 years	23	7.06%
**Number of brackets**		
0	150	46.01%
1	138	42.33%
≥2	38	11.66%
**Heart function classification**		
I	203	62.27%
II	120	36.81%
III	3	0.92%
**Smoking**		
Yes	125	38.34%
No	201	61.66%
**Drinking alcohol**		
Yes	68	20.86%
No	258	79.14%
**Left ventricular ejection fraction (%)**		
<50	20	6.13%
≥50	306	93.87%

### 3.2 The level of PA among young and middle-aged patients with CHD

Only 112 patients met the PA standards recommended by the European Society of Cardiology guidelines, accounting for 34.36% of the total.

### 3.3 Univariate analysis of PA among young and middle-aged patients with CHD

Univariate analysis revealed statistically significant differences in PA levels among young and middle-aged CHD patients with varying educational level, residence situation, monthly per capita household income, heart function classification, hemoglobin, glycated hemoglobin, fear of movement, exercise self-efficacy, family care, and fatigue (*P* < 0.05). No statistically significant differences were observed for the remaining factors (see Table 2).

**Table 2:** Univariate analysis of PA among young and middle-aged patients with CHD (*n* = 326).

**Variables**	**Qualified group (*n* = 112)**	**Non-qualified group (*n* = 214)**	***t*/*χ^2^*/*Z***	***P***
**Gender**				
Male	78	169	3.485	0.062
Female	34	45		
Age	55 (49, 58)	55 (47, 57)	−1.388	0.165
**Religious belief**				
Yes	1	4	–	0.664
No	111	210		
**Marital status**				
Unmarried	5	3	2.987	0.404
Married	103	201		
Divorce	3	7		
Widowed	1	3		
**Educational level**				
Primary school and below	9	17	−2.087	**0.037**
Junior high school	28	79		
High school/Technical secondary school	35	63		
Junior college	20	29		
Bachelor's degree or above	20	26		
**Residence situation**				
Living alone	5	24	4.134	**0.042**
Living with families	107	190		
**The location of the family**				
Rural area	11	10	−1.291	0.197
County town	8	16		
City	93	188		
**Monthly per capita household income (Yuan)**				
<2000	13	15	−3.445	**0.001**
2000–5000	25	117		
5001–10000	62	64		
>10000	12	18		
**Course of the disease**				
<1 year	34	65	−0.348	0.728
1–5 years	68	136		
>5 years	10	13		
**Number of brackets**				
0	49	101	−0.730	0.465
1	48	90		
≥2	15	23		
**Heart function classification**				
I	82	121	−3.003	**0.003**
II	30	90		
III	0	3		
Yes	50	75	2.864	0.091
No	62	139		
**Drinking alcohol**				
Yes	22	46	0.153	0.696
No	90	168		
Left ventricular ejection fraction (%)	54 (52, 57)	54 (51, 58)	−0.151	0.880
Hemoglobin (g/L)	133.74 ± 13.266	121 (110, 131.25)	−6.262	**0.000**
Albumin (g/L)	46.15 (40.10, 50.38)	45.45 (40.10, 51.10)	−0.105	0.917
Glycated hemoglobin (%)	5.30 (4.80, 5.70)	7.3 (5.3, 7.9)	−6.988	**0.000**
Fact-CAD	36.79 ± 7.70	42 (38.75, 47)	−6.589	**0.000**
ESES	58.89 (49.44, 69.72)	52.22 (43.89, 61.11)	−5.160	**0.000**
APGAR	8.0 (7.0, 9.0)	7.0 (7.0, 8.0)	−7.666	**0.000**
PFS-12	2.42 (2.17, 2.73)	2.92 (2.67, 3.17)	−8.729	**0.000**
PHQ-9	4.0 (3.0, 4.0)	4.0 (3.0, 4.0)	−0.508	0.612

Bold text indicates *P* < 0.05.

### 3.4 Binary logistic regression analysis of PA among young and middle-aged patients with CHD

Binary logistic regression analysis was performed with PA level as the dependent variable (qualified = 1, non-qualified = 0) and the 10 factors that showed statistical significance in the univariate analysis as independent variables. Detailed results of the final model are presented in Table 3. The analysis revealed that 6 of these 10 variables (hemoglobin, glycated hemoglobin, fear of movement, exercise self-efficacy, family care, and fatigue) were identified as independent predictors of PA levels among young and middle-aged patients with CHD (*P* < 0.05), while the remaining 4 variables included in the model showed no significant predictive effect (*P* > 0.05).

**Table 3:** Binary logistic regression analysis of PA among young and middle-aged patients with CHD (*n* = 326).

**Variables**	**β**	**SE**	**Wald χ^2^**	**OR (95%CI)**	***P***
Residence situation	−1.019	0.732	1.939	0.361 (0.086, 1.515)	0.164
Hemoglobin	0.033	0.014	5.755	1.034 (1.006, 1.062)	**0.016**
Glycated hemoglobin	−0.816	0.155	27.615	0.442 (0.326, 0.599)	**0.000**
Fact-CAD	−0.102	0.030	11.915	0.903 (0.852, 0.957)	**0.001**
ESES	0.051	0.020	6.531	1.052 (1.012, 1.093)	**0.011**
APGAR	0.848	0.227	13.941	2.334 (1.496, 3.642)	**0.000**
PFS-12	−3.177	0.553	33.005	0.042 (0.014, 0.123)	**0.000**
Educational level	0.002	0.189	0.000	1.002 (0.692, 1.451)	0.990
Monthly per capita household income	0.352	0.278	1.602	1.422 (0.824, 2.451)	0.206
Heart function classification	−0.785	0.426	3.407	0.456 (0.198, 1.050)	0.065

Bold text indicates *P* < 0.05.

Regarding the strength of influence among these 6 statistically significant independent predictors: Fatigue exerted the strongest negative effect on PA (OR = 0.042, 95%CI: 0.014–0.123, *P* < 0.001), followed by glycated hemoglobin (OR = 0.442, 95%CI: 0.326–0.599, *P* < 0.001). In contrast, family care had the strongest positive effect on PA (OR = 2.334, 95%CI: 1.496–3.642, *P* < 0.001), with exercise self-efficacy ranking second (OR = 1.052, 95%CI: 1.012–1.093, *P* = 0.011).

Additionally, a multicollinearity test was conducted for the final model. All independent variables included in the model had variance inflation factor values below 5 and tolerance values greater than 0.2, thus confirming the absence of significant multicollinearity. These findings confirm the stability and reliability of the final model results.

## 4 Discussion

This study confirmed that young and middle-aged patients with CHD generally exhibit low levels of PA in China, and its influencing factors include physiological, psychological, and social factors, which provides a basis for formulating targeted PA intervention strategies for this population. The above conclusion is supported by the following results: (1) Univariate analysis showed that there were significant differences in educational level, residence situation, monthly per capita household income, heart function classification, hemoglobin, glycated hemoglobin, fear of movement, exercise self-efficacy, family care, and fatigue between the PA qualified group and the non-qualified group (*P* < 0.05); (2) Multivariate logistic regression further confirmed that hemoglobin, glycated hemoglobin, fatigue, fear of movement, exercise self-efficacy, and family care were independent influencing factors of PA (*P* < 0.05).

### 4.1 The level of PA among young and middle-aged patients with CHD

This study found that a high proportion of 65.64% of young and middle-aged patients with CHD failed to meet the guideline-recommended standards for PA. This result is highly consistent with the research by Yao et al. (30), in which only 39.7% of patients achieved the recommended level, indicating that a similarly high proportion of 60.3% were also insufficiently active. These converging findings underscore the pressing need for clinical nursing practice to further promote the concept of exercise rehabilitation and provide systematic, scientific PA guidance for this population, so as to effectively increase their total activity volume and improve clinical outcomes.

### 4.2 Influencing factors of PA among young and middle-aged patients with CHD

#### 4.2.1 Physiological factors

Hemoglobin is a key protein in red blood cells responsible for transporting oxygen to tissues. A decrease in its level (i.e., anemia) can lead to a decline in oxygen-carrying capacity, making skeletal muscles more prone to hypoxia during exercise, thereby causing fatigue and breathing difficulties, and directly limiting the patient's exercise tolerance and PA levels. The study by Lipinski et al. (31) showed that hemoglobin levels were significantly and positively correlated with exercise tolerance. Specifically, patients with lower hemoglobin levels had significantly shorter total exercise time, lower achieved metabolic equivalents, and a lower maximum heart rate compared to those with higher hemoglobin levels. These findings strongly indicate that hemoglobin levels are a key determinant of a patient's exercise performance and a critical influencing factor for their PA levels. Therefore, in clinical practice, conducting routine hemoglobin screening for young and middle-aged patients with CHD and actively intervening for those with anemia may be one of the key links to improve their PA levels and promote the success of cardiac rehabilitation.

In addition to hemoglobin, glycated hemoglobin reflects a patient's average blood glucose level over the preceding 2 to 3 months, with elevated levels indicating poor glycaemic control. Persistent hyperglycaemia can impair muscle energy metabolism, thereby affecting PA. A study of older adult patients with type 2 diabetes demonstrated a significant association between elevated glycated hemoglobin and sarcopenia (32). The core mechanisms involve hyperglycaemia-exacerbated insulin resistance, chronic inflammation, and accumulation of advanced glycation end-products, collectively leading to reduced muscle protein synthesis and increased degradation. Although this study focused on older adults, the pathophysiological process whereby hyperglycaemia impairs muscle function is broadly applicable. Consequently, for young and middle-aged patients with CHD, elevated glycated hemoglobin levels similarly induce muscle energy metabolism disorders and premature fatigue through these mechanisms, thereby becoming a significant factor limiting their PA levels. Consequently, in clinical practice, glycated hemoglobin should be regarded not merely as a metabolic indicator but as an early warning signal for declining physical capacity. Concurrently, enhancing patient health education and self-management skills will increase their motivation to engage in exercise and adhere to treatment regimens.

Notably, fatigue itself, as a prevalent physiological symptom among CHD patients, also exerts a non-negligible impact on PA. A study showed that the fatigue levels of young and middle-aged patients with CHD were significantly higher than those of the general population (33). Long-term fatigue symptoms could reduce the patients' activity ability and increase the risk of complications from CHD (34). A prospective multicenter study of patients following coronary artery bypass graft surgery revealed that fatigue, as a significant physiological symptom, indirectly reduces PA levels through two pathways: increasing exercise phobia and diminishing self-efficacy (35). This indicates that fatigue is not merely a physiological impediment but also a catalyst triggering negative psychological responses, forming a mutual feedback loop that further restricts PA. In clinical practice, fatigue assessment should be incorporated into the rehabilitation monitoring of patients with CHD. Appropriate intervention measures should be developed based on the symptoms of fatigue. At the same time, psychological intervention should be coordinated to prevent fatigue from transforming into fear of movement and low exercise self-efficacy, and to break the negative cycle of the interaction between physiology and psychology.

#### 4.2.2 Psychological factors

Beyond physiological and metabolic factors, psychological elements also play a decisive role in patients' PA, with fear of movement being a frequently overlooked yet critical impediment. A qualitative research indicated that patients experiencing acute cardiovascular events often suffered from severe pain and a sense of near-death (36). They may worry about disease recurrence and the possibility of stent displacement during PA, which imposes a heavy psychological burden on themselves. These experiences can lead to negative emotions, including anxiety, depression, and grief, as well as fear of movement. Consequently, they may avoid PA, affecting both the initiation and maintenance of exercise. Research indicated that 20% of CHD patients exhibit severe kinesiophobia (37), rendering it a significant clinical concern. Consequently, clinical practice must incorporate the identification and intervention of fear of movement as an integral component of cardiac rehabilitation. Firstly, screening for fear of movement should be integrated into routine assessments to accurately identify high-risk individuals. Secondly, implement targeted interventions centered on cognitive behavioral education. This involves explaining the principles of cardiac rehabilitation to patients, clarifying that exercise within safe parameters protects rather than harms the heart. This helps patients overcome psychological barriers, enabling them to safely and effectively resume PA and achieve the ultimate goal of cardiac rehabilitation.

In addition to fear of movement, exercise self-efficacy is another core psychological factor affecting PA persistence. This study showed that exercise self-efficacy is an independent influencing factor for PA. Exercise self-efficacy, as the specific manifestation of self-efficacy within the sporting domain, serves as a crucial psychological indicator for predicting the persistence of exercise behavior (38). A study revealed a significant positive correlation between exercise self-efficacy and PA levels in patients following acute type A aortic dissection surgery; that is, higher exercise self-efficacy scores were associated with greater PA levels (39). Consistently, a survey of 149 discharged patients with CHD confirmed this positive relationship. Multivariate regression analysis further showed that exercise self-efficacy was an independent predictor of PA, which was consistent with the results of this study (40).

Overall, fear of movement and exercise self-efficacy influence PA through a “hindrance-promotion” dual pathway. In the future, joint intervention studies can be conducted to verify the improvement effect of the dual-target intervention of “fear of movement screening and self-efficacy enhancement” on PA.

#### 4.2.3 Social factors

Beyond intrinsic physiological and psychological factors, the external social environment also plays a crucial role in regulating PA levels of young and middle-aged patients with CHD, among which family care is the most direct and important social support factor. As the core social support system for patients, family care exerts a positive regulatory effect on PA through dual pathways of emotional support and practical supervision. Specifically, family members can provide emotional encouragement to alleviate patients' anxiety about PA, accompany patients to participate in rehabilitation exercise to enhance their sense of security, and supervise the implementation of exercise plans to improve adherence. The results of this study were consistent with previous studies. Previous research pointed out that family support was a predictor of adherence to cardiac rehabilitation, and patients with stronger family support had a higher persistence in exercise plans (41). A study that included 378 middle-aged and older adult patients with stable CHD also confirmed that social support was an important influencing factor for PA (30), which was consistent with the positive regulatory effect of family care on PA in this study, further confirming the core value of family care in PA intervention for patients with CHD.

The results of this study have clear guiding significance for clinical practice and patient care. In clinical work, the assessment of family care can be incorporated into the discharge rehabilitation assessment system for young and middle-aged patients with CHD. For patients with insufficient family support, a family participation-based intervention plan can be developed—such as guiding family members to master basic rehabilitation exercise knowledge, jointly formulating personalized exercise plans, and establishing a regular follow-up mechanism to urge family members to fulfill their responsibilities of accompanying and supervising, in order to strengthen the promoting effect of family support on PA.

## 5 Limitation

This study used IPAQ-L to assess PA, which relies on the respondents' recall of past activities, and there may be recall bias. Although we took measures such as guided recall and outlier screening to reduce bias, it cannot be completely eliminated. In future studies, objective measurement tools (such as accelerometers) can be used to assess PA to improve the accuracy of data.

## 6 Conclusion

This study revealed that young and middle-aged patients with CHD generally exhibit low levels of PA, a situation resulting from the combined influence of physiological, psychological, and social factors. Patients' PA levels correlated with hemoglobin, glycated hemoglobin, fear of movement, exercise self-efficacy, family care, and fatigue. In clinical practice, healthcare professionals should conduct comprehensive assessments of patients' physiological, psychological, and social conditions to develop personalized PA intervention plans, thereby promoting cardiac rehabilitation.

## Data Availability

The original contributions presented in the study are included in the article/supplementary material, further inquiries can be directed to the corresponding author.
